# Recent advances in the biology of human circulating tumour cells and metastasis

**DOI:** 10.1136/esmoopen-2016-000078

**Published:** 2016-08-03

**Authors:** Sofia Gkountela, Barbara Szczerba, Cinzia Donato, Nicola Aceto

**Affiliations:** Cancer Metastasis, Department of Biomedicine, University of Basel, Basel, Switzerland

**Keywords:** circulating tumor cells, metastasis

## Abstract

The development of a metastatic disease is recognised as the cause of death of over 90% of patients diagnosed with cancer. Understanding the biological features of metastasis has been hampered for a long time by the difficulties to study widespread cancerous lesions in patients, and by the absence of reliable methods to isolate viable metastatic cells during disease progression. These difficulties negatively impact on our ability to develop new agents that are tailored to block the spread of cancer. Yet, recent advances in specialised devices for the isolation of circulating tumour cells (CTCs), hand-in-hand with technologies that enable single cell resolution interrogation of their genome and transcriptome, are now paving the way to understanding those molecular mechanisms that drive the formation of metastasis. In this review, we aim to summarise some of the latest discoveries in CTC biology in the context of several types of cancer, and to highlight those findings that have a potential to improve the clinical management of patients with metastatic cancer.

## Introduction

Despite remarkable improvements in early detection of cancerous lesions, combined with surgical removal and treatment of the primary tumour, the development of a metastatic disease remains the main cause of death for the vast majority of patients with cancer. Currently, worldwide, more than seven million people per year die as a consequence of a metastatic disease (WHO). Clearly, these numbers reflect our limited understanding of the biology of cancer in the metastatic setting, and the need to overcome a number of clinical as well as technical limitations to better comprehend how to efficiently target metastasis in patients.

Clinically, patients with a metastatic disease are often characterised by the presence of one or more micrometastatic and macrometastatic foci throughout various organs.^1^ Only some of these foci are clinically detectable at the time of metastasis diagnosis, while many more foci are likely to be present but below detection limit, yet posing a significant risk in terms of disease progression.^1^ ^2^ In this context, treatment strategies for patients with metastasis are often based on the pathological and molecular characterisation of the primary tumour, while little or no information is available from the various metastatic lesions that are present at that specific moment. A main reason for this is that metastases are hardly accessible for direct sampling. However, the consequence is that we still treat metastasis based on information obtained from a primary tumour, an approach that has led to no success. Further, we now started to be aware of the fact that metastasis is an evolutionary process, where metastatic subclones with a unique mutational profile may emerge along with cancer progression at any time, resulting in a high degree of heterogeneity and significant differences from the primary tumour of origin.^3^

Technically, our current understanding of cancer heterogeneity, especially in the metastatic setting, argues that even if we were able to biopsy most of the metastatic lesion in a given patient, we would still face the issue of cellular heterogeneity within each lesion.^4^ However, newly established protocols now enable a single cell resolution interrogation of the genome and transcriptome of cancer cells, and their application to the metastasis field holds the promise to define its molecular drivers with high precision.^5^

While metastasis remains very challenging for a direct biopsy, recent developments in microfluidics technologies are enabling the capture of live circulating tumour cells (CTCs) from the blood of patients with various types of cancer.^6^ Most excitingly, given the short half-life of CTCs in circulation,^7^ in the metastatic setting CTCs are a direct derivative of metastasis. Pragmatically, they can be seen as an opportunity to isolate, in real time, live cancer cells that are derived from proliferating metastatic lesions in patients, thereby empowering a single cell resolution analysis of metastasis from a minimally invasive liquid biopsy.

It is important to mention that CTCs are extraordinarily rare in the blood of patients with cancer (on average, one CTC per billion normal blood cells), even in those patients with progressing metastatic disease.^8^ In fact, detection of CTCs greatly varies depending on the technology used for their isolation. The majority of platforms currently available for CTC isolation rely on the expression of cell surface markers or physical properties to distinguish CTCs from normal blood components.^9–15^ For epithelial cancer types that generally express high levels of epithelial cell adhesion molecule (EpCAM), the CellSearch system is currently the only Food and Drug Administration (FDA)-approved device in the clinical setting.^12^ ^13^ ^16^ On the other hand, size-based enrichment platforms, such as Parsortix and ScreenCell, take advantage of the slightly bigger size of CTCs compared with red and white blood cells (WBCs; ∼12–25 μm for a single CTC vs 8 and 7–15 μm for a red and WBC, respectively).^17–19^ Key to CTC enumeration and characterisation are also newly developed microfluidic devices, such as the spiral biochip based on hydrodynamic forces, or the CTC-iChip which uses a combination of hydrodynamic cell separation and immunomagnetic depletion of antibody-tagged WBCs to isolate larger CTCs.^15^ ^20^ However, an unbiased assessment of CTC number and molecular characteristics still requires to overcome a very high degree of technical difficulties, reviewed elsewhere, such as the need for antigen-independent CTC enrichment techniques,^14^ devices for single cell manipulation with minimal losses,^21^ ^22^ DNA and RNA amplification protocols for single cells with low sequence biases^23^ and bioinformatics tools that facilitate single cell data normalisation and analyisis.^24^ These technologies are now within reach, and are likely to enable a comprehensive characterisation of metastatic cells in the near future.

In this review, we aim to summarise some of the most significant discoveries in CTC biology in different cancer types, focusing on those that are likely to impact our understanding of the metastatic process.

## The biology of CTCs in different cancer types

The analysis of CTCS in several cancer types has already allowed a better understanding of the metastatic process, as well as it has highlighted the use of CTCs as a non-invasive source of information for individualised medicine. However, much work remains to be done to gain those insights that will allow the development of new metastasis-tailored therapies. While most of our understanding of CTC biology derives from the analysis of breast and prostate CTCs, recently we also witnessed important advances in colorectal, pancreatic and lung cancer, as well as in melanoma and glioblastoma multiforme (GBM).

### Breast cancer

Breast cancer is the second most common cancer in the world, and by far the most common cancer in women, with more than 1.6 million new cases each year. A vast proportion of breast cancers are cured with surgery; however, the development of metastasis still accounts for more than 500 000 deaths per year worldwide (WHO).

Breast cancer is the cancer in which most studies related to CTCs have been carried out. For example, breast CTCs have been shown to be predictors of decreased survival in patients with early breast cancer, before or after chemotherapy, with prognosis being worst in those patients with at least five CTCs per 30 mL of blood.^25^ Along these lines, systemic spread of breast cancer has been shown to start early during tumour progression in patients and mouse models, where CTCs are released from a growing primary tumour mass and disseminate to distant organs, leading to the development of metastasis.^26^ In the metastatic setting, high CTC counts have also been shown to be predictive of bad prognosis, including those patients who were newly diagnosed with metastatic breast cancer and were about to start first-line systemic treatment.^27^ ^28^

One of the key aspects that emerged from the analysis of breast CTCs is their remarkable heterogeneity, both considering the expression of specific cancer-associated markers, and also their phenotypic characteristics such as tumour-seeding potential. For instance, CTCs frequently lack estrogen receptor (ER) expression in patients with metastatic breast cancer who were diagnosed with ER-positive primary tumours, and these CTCs show a high degree of intrapatient heterogeneity which may reflect a mechanism to escape endocrine therapy.^29^ ^30^ Similarly, the expression of the human epidermal growth factor receptor 2 (HER2) on CTCs is often not concordant with the HER2 status of the primary tumour, and a subset of patients with HER2-negative primary tumour will develop HER2-positive CTCs during disease progression.^31^ These findings have clear implications for what concerns targeted therapy approaches, and highlight the need to characterise CTCs in real time to define the best treatment for individual patients.

In terms of phenotypic characteristics, significant efforts have been conducted on breast CTCs to identify mesenchymal-like cells among them, and also determine which CTCs in a patient are more likely to successfully form metastasis at a distant site. Epithelial-to-mesenchymal transition (EMT) has been observed in human CTCs from patients with breast cancer, highlighting the occurrence of mesenchymal-like CTCs during disease progression.^8^ In an index patient who received longitudinal blood monitoring for epithelial and mesenchymal CTCs along treatment, reversible shifts between these cell fates accompanied each cycle of response to therapy and disease progression, suggesting that EMT may occur as a consequence to treatment failure.^8^ Although EMT has now been shown to occur in human specimens, it is important to highlight that the requirement of EMT for the development of metastasis is still highly controversial.^32^ ^33^

A clinically relevant question regarding CTC biology is whether dissecting CTC heterogeneity in breast cancer can help to identify CTC populations with increased tumour-seeding potential. In this regard, it was recently shown that in patients with breast cancer, the metastasis initiating CTCs were confined in a subpopulation of EpCAM-positive CTCs that also expressed CD44, CD47 and the tyrosine kinase receptor c-MET.^34^ This finding was supported by evidence in mouse xenograft models, and based on the increased ability of EpCAM^+^/CD44^+^/CD47^+^/c-MET^+^ CTCs to form bone, lung and liver metastases when transplanted in the femoral cavity of immunocompromised mice, compared with the entire population of EpCAM-expressing CTCs.^34^ These metastasis-initiating markers were also expressed in patient metastases as judged by histological assessment.^34^ Interestingly, another recent study identified a brain metastasis expression signature in the EpCAM-negative fraction of breast CTCs, defined as a subpopulation of HER2^+^/EGFR^+^/HPSE^+^/Notch1^+^ CTCs.^35^ These results indicate that multiple subpopulations of metastasis-initiating CTCs can be present in the peripheral blood of patients with breast cancer.

In addition to markers, certain physical properties of CTCs were also recently linked to increased metastatic potential. In mouse xenograft models, CTC clusters were shown to carry a 23-fold to 50-fold increased metastatic potential compared with single CTCs.^7^ In patients with breast cancer, the presence of CTC clusters is also associated with decreased metastasis-free survival and the development of new metastatic foci.^7^ Interestingly, these CTC clusters were shown to arise from oligoclonal expansion of tumour cell groupings, rather than from the aggregation of single CTCs in the vasculature or the proliferation of a single CTC. Detailed molecular profiling of single and clustered CTCs from patients with metastatic breast cancer identified the cell–cell junction component plakoglobin to be required for CTC cluster formation. Further, plakoglobin expression in the primary tumour of patients with breast cancer is associated with a reduced metastasis-free survival.^7^ In a separate study, CTC clusters were also shown to have an increased metastatic potential and to express keratin14, a marker that was previously found expressed in basal breast cells.^36^ Additionally, CTC clusters are found in some instances in association to WBCs, platelets and fibroblasts, which in turn may promote survival and shape the molecular profile of the cells they are in contact with, further promoting heterogeneity and tumour-seeding potential.^7^ ^37–39^ These results highlight CTC clusters as extraordinarily efficient metastatic precursors in breast cancer, and the need to further refine their characterisation in breast and other cancer types, to identify their key vulnerabilities.

### Prostate cancer

Prostate cancer is the second most common cancer in men, with ∼1.1 million new cases each year worldwide, leading to more than 300 000 deaths per year (WHO). Patients with prostate cancer succumb to this disease because of the development of a metastatic disease that is resistant to therapeutic agents, and that usually involves the bone as the primary metastatic site.^40^

In advanced prostate cancer, similarly to breast cancer, CTC enumeration has been used as a prognostic tool for disease progression and survival;^41^ however, in localised prostate cancer, a clear correlation between CTC numbers and clinical outcome has not been found.^42^ ^43^

More generally, prostate cancer is frequently responsive to androgen deprivation therapy, given the high expression and requirement of the androgen receptor (AR) in the development and progression of this disease.^40^ However, the effectiveness of AR inhibitors in recurrent metastatic disease is highly variable. While direct sampling of metastatic lesions in these patients is challenging, CTC analysis may be key to reveal the mechanisms of innate or acquired resistance to AR-targeted therapies. For this reason, most recent studies have been focusing on performing detailed molecular characterisation of CTCs isolated from patients with metastatic prostate cancer. For example, whole exome sequencing of CTCs was employed to detect somatic single nucleotide variants in patients with prostate cancer.^44^ In this study, the mutational profile of CTCs was compared with that of the primary tumour and metastasis and showed a high degree of similarity, establishing a proof-of-concept that CTC genomics can be used in the clinic as a non-invasive method to assess the mutational landscape of metastatic prostate cancer.^44^ In a similar approach, whole genome amplification of CTCs isolated from castration-resistant patients with prostate cancer was used to assess copy number aberrations.^45^ In this study, the majority of aberrations found in CTCs were also present in the primary tumour; however, copy number gains at the AR locus were found specifically in CTCs, arguing that they could have emerged as a consequence of AR therapy resistance.^45^

In a recent study that employed single cell resolution RNA sequencing of CTCs, putative stem cell markers, such as ALDH7A1, CD44 and KLF4, as well as markers for cell proliferation were enriched in prostate CTCs compared with primary tumours.^46^ Of note, in this study, single prostate CTCs displayed a significant heterogeneity, including the expression of AR gene mutations and splicing variants, such as AR-v7, previously shown to confer resistance to antiandrogen therapies.^46^ ^47^ Retrospective analysis of CTCs from patients who were progressing under AR-targeted treatment, compared with untreated cases, showed a remarkable activation of non-canonical Wnt signalling pathway. Ectopic expression of Wnt5a in prostate cancer cells attenuated the antiproliferative effects of AR inhibition, whereas its suppression in drug-resistant cells restored partial sensitivity to anti-AR treatment.^46^ Altogether, these studies provide evidence that CTC analysis in patients with metastatic prostate cancer is an opportunity to reveal those key mechanisms of resistance to AR inhibitors, as well as to identify those patients who would benefit the most from a targeted treatment.

### Colorectal cancer

Colorectal cancer (CRC) is the second most common cancer in women and the third most common cancer in men, with a total of more than 1.3 million new cases each year. Owing to the development of a metastatic disease, usually affecting the liver at first, almost 700 000 patients succumb to this disease yearly worldwide (WHO).

CTCs have been observed in patients with CRC, in the metastatic as well as non-metastatic setting.^48–50^ Notably, the number of CTCs in CRC was also shown to be important in predicting the development of tumour recurrence and emergence of distant metastasis.^48^ ^51–53^ More generally however, the number of CTCs detected in the peripheral blood of patients with CRC is much lower compared with other cancers such as breast cancer. Along this line, a recent study compared CTC numbers in blood drawn from peripheral or mesenteric blood in patients with CRC, and found a higher number of CTCs in mesenteric blood samples (median of 2.7–4 CTCs in mesenteric blood vs 0–2 CTCs in peripheral blood), indicating that a considerable portion of CTCs are likely to be trapped in the liver before they reach the peripheral circulation, as the liver is the first filter organ that CTC will encounter on release from the primary tumour mass in the colon.^54^ Thus, CTC isolation efforts should also take into account the site of the primary tumour mass (and eventually of each of the metastatic deposits) as well as the blood circulation dynamics to efficiently capture the most viable cancer cells in circulation.

CTCs from patients with metastatic CRC have also been the first to be sequenced at the single cell level. Single cell array comparative genomic hybridization (CGH) and parallel sequencing of 68 CRC-associated genes confirmed the presence of driver mutations in genes such as APC, KRAS or PIK3CA in the primary tumour, metastasis and corresponding CTCs from two patients.^55^ However, certain mutations were only visible in CTCs, probably due to their low frequency in the primary tumour and metastatic deposits.^55^ These results suggest that liquid biopsy in patients with CRC is highly promising strategy to monitor tumour genomes in real time and facilitate personalised therapy.

### Pancreatic cancer

Pancreatic ductal adenocarcinoma (PDAC) is diagnosed in more than 300 000 individuals each year (World Cancer Research Fund, WCRF). Owing to its highly aggressive nature and the fact that early stages of this cancer do not usually produce symptoms, PDAC is almost always fatal, with a 5-year survival rate at ∼5% (WCRF). The development of metastasis is the main cause of death in patients with this disease.

Patients with PDAC have been examined for the presence of CTCs in their bloodstream, which were detected with various technologies and at various concentrations.^56–59^ However, individually, these studies failed to find a clear correlation between CTC abundance and prognosis. Taken together into a meta-analysis on 623 patients, CTC-positive patients with PDAC showed a worse prognosis compared with patients with no detectable CTCs, independently from the detection method that was used.^60^

Alterations in the gene encoding KRAS are a key feature in PDAC, and KRAS mutations have been accordingly found in PDAC CTCs.^61^ Along the line of a molecular characterisation of PDAC CTCs, single cell resolution RNA sequencing of human and mouse PDAC CTCs has highlighted the expression of extracellular matrix (ECM) genes in these cells,^59^ as well as the activation of Wnt signalling.^62^ In these studies, mouse PDAC CTCs showed upregulation of Wnt2,^62^ low-proliferative signatures, enrichment of the stem cell-associated gene ALDH1A2, a biphenotypic expression of epithelial and mesenchymal markers and the expression of IGFBP5, a gene transcript enriched at the epithelial–stromal interface in PDAC primary tumours.^59^ Yet, both mouse and human PDAC CTCs displayed a very high expression of stromal-derived ECM proteins, including SPARC, whose knockdown in cancer cells suppressed cell migration and invasiveness.^59^ These results highlight that PDAC CTCs may employ expression of Wnt signalling effectors and ECM proteins to facilitate their *route* to metastasis. However, further studies are required to identify effective therapeutic targets with potential to suppress the spread of PDAC cells.

### Lung cancer

Lung cancer has been the most common cancer in the world for several decades, with ∼1.8 million new cases diagnosed each year (WHO). Lung cancer is also the most common cause of cancer-related death, with ∼1.6 million deaths worldwide due to this disease, yearly.

Lung cancer is a key example in targeted therapy approaches, since patients with non-small-cell lung cancer (NSCLC) harbouring activating mutations in the EGFR gene demonstrate a significant progression-free survival benefit when treated with EGFR tyrosine kinase inhibitors (TKIs).^63^ However, the majority of patients that are initially responding will develop acquired resistance after 12–24 months of treatment. Mechanisms to TKI resistance include the development of a recurrent T790M EGFR mutation, amplification of signalling molecules that bypass EGFR inhibition (such as MET and HER2), mutations in other oncogenic drivers (eg, PIK3CA and B-RAF) and conversion to small-cell lung cancer (SCLC).^64–69^ In this context, the possibility to interrogate lung cancer genotype in real time through liquid biopsies is of paramount importance. In patients with EGFR-mutant NSCLS, it was previously shown that an allele-specific assay was able to detect the emergence of T790M mutations in CTCs during first-line therapy.^70^ Subsequently, other studies confirmed that the analysis of lung CTCs can enable the monitoring of evolving tumour genotype in some patients.^71–74^ In addition to their genotype, physical characteristics of NSCLC CTCs have been studied, revealing that NSCLC CTCs appear as single or clustered, with the latter being mostly negative for the proliferation marker Ki67.^75^

In SCLS, CTCs have been detected in great numbers and their abundance clearly correlates with a reduced overall survival.^76^ More specifically, patients with more than 50 CTCs per 7.5 mL of blood have an overall survival of 5.4 months, compared with patients with <50 CTCs per 7.5 mL of blood, characterised by an overall survival of 11.5 months.^76^ CTCs in SCLC are detected as both single CTCs and CTC clusters, with the latter appearing protected from anoikis and with an increased resistance to cytotoxic drugs.^76^ Interestingly, CTCs from patients with SCLC have been also recently employed for transplantation in immunocompromised mice, thus recapitulating the features of the tumour growing in the donor patient.^77^ In this model, genomic analysis of the CTC-derived xenografts revealed a high degree of similarity to the original tumour, and a similar responsiveness to platinum and etoposide chemotherapy,^77^ thereby providing an excellent platform to guide precision medicine.

### Melanoma

Melanoma is diagnosed in more than 230 000 patients per year worldwide, and approximately one-fifth of these patients are lost each year (WCRF). The main cause of death in patients with melanoma is the development of a systemic metastatic disease, affecting most frequently organs such as the liver, bone and brain.^78^

In the past 5 years however, a paradigm shift has occurred in the treatment of this disease. First, a better understanding of the genetic landscape of melanoma has allowed the development of targeted therapies with efficacy against this disease. One above all, is the discovery that B-RAF oncogene is mutated in ∼50% of melanomas, and that patients with this genotype benefit from therapy with B-RAF and MEK inhibitors,^79–81^ although most will develop resistance within 12 months.^82–84^ Second, the understanding of key pathways controlling the immune system has led to the development of immune checkpoint inhibitors such as antibody antagonists of CTLA-4 and PD-1, which individually confer a significant survival benefit to a subset of patients, and even better responses when combined.^85–89^ However, at present it is still unclear which patients will benefit from these agents, therefore the identification of biomarkers of response is a priority. More specifically, in melanoma, acquired resistance to therapy seems to be driven by the clonal expansion of resistant tumour cells.^90^ While repeated biopsies to study genomic alterations along therapy are invasive, difficult to obtain and prone to be confounded by intratumoural heterogeneity, the analysis of CTCs may result as a powerful weapon to stratify patients in light of the best treatment option.

Since melanoma is not an epithelial cancer, CTCs are extraordinarily hard to isolate from the blood of patients, because they do not express common CTC markers such as EpCAM or epithelial cytokeratins that would distinguish them from normal blood cells. However, their isolation is now achievable through antigen-agnostic techniques.^14^ This is enabling a better understanding of their biology, and highlighting the possibility to stratify patients before treatment. Melanoma CTC xenografts have been also recently developed, and hold the promise to serve as a platform to screen various therapeutic agents in vivo, while gaining insights into tumour evolution dynamics.^91^ Further, melanoma CTCs have been detected in patients along B-RAF-targeted therapy, with their number increasing during disease progression.^92^ Lastly, a recent report has shown that the number of melanoma CTCs in patients is much higher when blood is taken from the arterial, rather than venous, circulation.^93^ Along the lines of what is discussed above for CRC, these results indicate a better, although rather inconvenient for the patient, source of blood sampling for melanoma CTCs. Altogether, these studies demonstrate the ability to isolate and characterise melanoma CTCs from patients, and may be key in the future to achieve patient stratification before the administration of targeted therapy or immunotherapy.

### Glioblastoma multiforme

Worldwide, there are an estimated 240 000 cases of brain and nervous system tumours per year, with GBM being the most common, and the most lethal, of these tumours (WHO). Unlike other tumours, patients with GBM die because of the consequences of tumour growth in the primary tumour site, and development of metastasis is extremely rare.^94^

Major challenges in the treatment of GBM include the inability to excise tumour cells infiltrating into normal brain tissue, the poor penetration of therapeutic agents into the central nervous system (CNS), the difficulty in distinguishing tumour responses from recurrence using standard imaging criteria and the inherent risks associated with brain biopsies needed to monitor tumour evolution during disease progression.^94^ In this context, it has been clear for long time that the possibility to isolate GBM CTCs from liquid biopsies may significantly help in understanding GBM biology. However, until very recently, it has been unclear whether GBM cancer cells would be able to cross the blood–brain barrier and be detectable in the peripheral circulation. Further, similarly to melanoma and unlike many epithelial cancer types, CNS malignancies do not express EpCAM, a marker that is commonly used for CTC detection. Thus, the isolation of GBM CTCs has been hampered by a number of exceptionally hard challenges.

However, in 2014, three groups reported the successful isolation and characterisation of CTCs from the peripheral blood of patients with GBM.^95–97^ In a first study, glial fibrillary acidic protein-expressing CTCs were detected in 29/141 patients with GBM.^95^ These CTCs were identified in the density gradient purified mononuclear fraction of peripheral blood and further validation was based on the expression of EGFR mutations and aberrations that matched the primary GBM tumours. However, there was no correlation between CTC enumeration and clinical outcome before or after surgery in these patients.^95^ In a second study, using a strategy based on telomerase activity, GBM CTCs were successfully detected in a number of patients.^96^ Specifically, CTCs were detected in 8/11 preradiotherapy patients as opposed to 1/8 in postradiotherapy patients, indicating that CTC enumeration in GBM could be useful in identifying patients who are at high risk of recurrence.^96^ In the third study, using the CTC-iChip platform combined with a specific staining optimised to distinguish GBM cells from any other blood cell, CTCs were found in 13/33 patients with GBM.^97^ These CTCs were identified using a cocktail of fluorescent probes targeting five known high-grade glioma markers, termed ‘STEAM’ (SOX2, Tubulin β-3, EGFR, A2B5, c-MET).^97^ As further validation, CTCs were shown to harbour EGFR gene amplifications that corresponded to the primary GBM tumour. Additional single cell resolution expression analyses identified a high enrichment of mesenchymal-associated transcripts, such as SERPINE1, TGFB1, TGFB2 and vimentin at the expense of neural lineage markers, compared with matched primary tumours. Interestingly, these mesenchymal markers were also expressed by RNA-ISH at distinct areas of the primary tumour and predominantly at the invasive edge of the deep white matter tracts, the area of the tumour that is associated with GBM cell invasion.^97^ Nevertheless, in this study there was no clear correlation between CTC presence and clinical outcome of these patients.^97^ Altogether, these studies were instrumental to demonstrate for the first time the presence of CTCs in patients with GBM, and warrant further investigations to gain more insights into the biology of this disease.

### Culturing CTCs for personalised medicine

While the analysis of freshly isolated CTCs might be a phenomenal opportunity to stratify patients and to guide precision medicine in the future, the extremely low abundance of these cells in the peripheral blood of patients with cancer remains a challenge in the context of personalised drug screenings. The possibility of expanding CTCs in culture has only very recently been achieved, carrying important implications for personalised medicine.

The first study reporting successful culture of CTCs was performed with samples from patients affected by brain metastatic breast cancer.^35^ In this study, a fraction of EpCAM-negative CTCs was found to carry a HER2/EGFR/HPSE/Notch1 protein signature and to be particularly prone to form brain metastasis. These cells were cultured, and on transplantation in mice, these CTC cell lines were highly invasive and capable to generate brain and lung metastasis in animal models.^35^ However, the first example of CTC cultures with the aim of individualised drug testing was provided in a different study.^98^ In that study oligoclonal CTC cultures were derived from six patients with ER-positive breast cancer, and subjected to genome sequencing of a panel of cancer-associated mutational hotspots. Data analysis revealed pre-existing as well as acquired mutations in PIK3CA, ESR1 and FGFR2 genes, among others. Drug sensitivity testing ex vivo and xenografts of each CTC cell line revealed key (personalised) vulnerabilities as a proof-of-concept.^98^ In another study, CTCs as well as tumour biopsies derived from patients with prostate cancer were expanded as long-term organoid cultures.^99^ Seven newly generated organoid cell lines were shown to recapitulate the molecular diversity of prostate cancer subtypes, including TMPRSS2-ERG fusion, SPOP, FOXA1 and PIK3R1 mutations, SPINK1 overexpression, and loss of CHD1, p53 and RB tumour suppressor.^99^ Other studies that followed could then show for the first time a successful CTC culture establishment from CRC^100^ and lung cancer,^101^ paving the way to a detailed molecular and phenotypic analysis of CTCs in these diseases as well.

Altogether, several groups have now successfully established long-term CTC cultures from different cancer types. In the context of personalised medicine however, much work remains to be done. For instance, establishment of CTC-derived cell lines nowadays still requires several months, and it is only possible from a restricted number of patients, usually those with the highest numbers of CTCs.^98–101^ During this time, most CTCs isolated from a patient will die in culture, and only some will be able to successfully grow and establish a cell line. During this process, the corresponding patient in the clinic is likely to undergo additional treatment cycles, which are expected to reshape the molecular portrait of his/her disease.^102^ ^103^ In this scenario, a drug screening on CTC-derived cells would not be up to date with the patient's disease. For CTC cultures to become a strategy that enables real-time personalised medicine, much progress needs to be made in order to achieve drug susceptibility testing within only a few weeks, if not just days, after the blood is drawn. Thus, increasing the success rates of CTC culture assays along with the development of more rapid culture strategies is of paramount importance for achieving personalised drug screenings from liquid biopsy, as well as to enable most patients with cancer to benefit from this approach.

## Conclusions

In the past few years, the CTC field has witnessed outstanding advances. First, it is now possible to efficiently isolate CTCs in an antigen-agnostic fashion.^7^ ^15^ ^18^ ^20^ This allows an unbiased CTC enrichment strategy in epithelial cancers, and also permits the isolation of CTCs from cancers of non-epithelial origin, such as GBM and melanoma. Second, several CTC-enrichment technologies are now able to release viable CTCs in solution, thereby empowering their micromanipulation or culture, and separation from contaminant blood cells after first-step enrichment.^7^ ^46^ ^98^ Third, single cell resolution sequencing of the genome or transcriptome of CTCs has been achieved,^5^ showing that it may represent an extraordinary opportunity to characterise the mutational profile of metastatic cells in real time, to interrogate patient samples longitudinally during treatment, as well as to dissect fundamental pathways that orchestrate the metastatic process. Fourth, several groups have shown the ability to expand CTCs in culture or as xenografts with the goal of testing individualised drug susceptibility, and creating new CTC-derived lines that represent highly clinically relevant models to study how metastasis occurs at the molecular level.^98–101^

These extraordinary discoveries in the CTC field, however, should be seen as the starting point of a journey that promises to bring liquid biopsies into clinical practice. Significant steps ahead are urgently needed to achieve standardised protocols for real-time CTC monitoring and molecular interrogation, early during primary tumour onset and also later during metastatic disease progression, most likely in conjunction with the analysis of cell-free DNA in patients with cancer.^104^ At the same time, CTC culturing needs to be achieved in a much faster time frame in order to benefit patients. Last but not least, several outstanding questions remain unanswered in the CTC and metastasis fields. For example, we still do not know what triggers the generation of CTCs (single or clustered) from a primary tumour or metastatic deposit, what is the true evolution pattern of metastasis at the single cell level before and after therapy and what are the targets to inhibit in order to prevent or suppress the haematogenous spread of cancer cells in patients. Answers to these questions are now within reach, and hold great promise to improve the clinical management of patients who suffer from metastatic cancers.
